# The impact of magnesium content on lithium-magnesium alloy electrode performance with argyrodite solid electrolyte

**DOI:** 10.1038/s41467-024-48071-0

**Published:** 2024-05-27

**Authors:** Jack Aspinall, Krishnakanth Sada, Hua Guo, Souhardh Kotakadi, Sudarshan Narayanan, Yvonne Chart, Ben Jagger, Emily Milan, Laurence Brassart, David Armstrong, Mauro Pasta

**Affiliations:** 1 Department of Materials, University of Oxford, Parks Road, Oxford, OX1 3PH UK; 2 The Faraday Institution, Harwell Campus, Quad One, Becquerel Avenue, Didcot, OX11 0RA UK; 3 Department of Sustainable Energy Engineering, Indian Institute of Technology, Kanpur, 208016 India; 4 Department of Engineering Science, University of Oxford, Parks Road, Oxford, OX1 3PJ UK

**Keywords:** Batteries, Energy storage, Materials for energy and catalysis, Electrochemistry

## Abstract

Solid-state lithium-based batteries offer higher energy density than their Li-ion counterparts. Yet they are limited in terms of negative electrode discharge performance and require high stack pressure during operation. To circumvent these issues, we propose the use of lithium-rich magnesium alloys as suitable negative electrodes in combination with Li_6_PS_5_Cl solid-state electrolyte. We synthesise and characterise lithium-rich magnesium alloys, quantifying the changes in mechanical properties, transport, and surface chemistry that impact electrochemical performance. Increases in hardness, stiffness, adhesion, and resistance to creep are quantified by nanoindentation as a function of magnesium content. A decrease in diffusivity is quantified with ^6^Li pulsed field gradient nuclear magnetic resonance, and only a small increase in interfacial impedance due to the presence of magnesium is identified by electrochemical impedance spectroscopy which is correlated with x-ray photoelectron spectroscopy. The addition of magnesium aids contact retention on discharge, but this must be balanced against a decrease in lithium diffusivity. We demonstrate via electrochemical testing of symmetric cells at 2.5 MPa and 30^∘^C that 1% magnesium content in the alloy increases the stripping capacity compared to both pure lithium and higher magnesium content alloys by balancing these effects.

## Abstract (web summary)

All-solid-state lithium-based batteries require high stack pressure during operation. Here, we investigate the mechanical, transport, and interfacial properties of Li-rich magnesium alloy and show enhanced performance at low pressure and ambient temperature for alloy-based electrodes with less than 5 at% Mg content.

## Introduction

Solid-state battery (SSB) technology incorporating inorganic solid-state electrolytes is an attractive option to power electric vehicles (EVs), primarily as it could enable the safe implementation of lithium metal negative electrodes (theoretical capacity ~ 3860 mAh ⋅ g^−1^ and 2061 mAh ⋅ cm^−3^)^1^. These can lead to cells with gravimetric and volumetric energies upwards of 400 Wh ⋅ kg^−1^ and 1000 Wh ⋅ L^−1^, which are thermally stable and amenable to fast charging^2^.

Unfortunately, pure lithium metal negative electrodes struggle to meet the performance requirements of SSBs for EVs. This is due to lithium’s material properties. Contact loss with the electrolyte is observed on stripping^3^, which is often attributed to slow diffusion kinetics and insufficient creep^4^. After repeated Li plating on the negative electrode, the current becomes concentrated at the remaining contact area leading to lithium filament formation^5^. Due to lithium’s high nanoscale hardness^6,7^, these filaments propagate through cracks and drive electrolyte fracture^8,9^. At the interface with the solid electrolyte, lithium’s high reactivity leads to detrimental surface reactions^10^ forming a solid electrolyte interphase (SEI) which can adversely impact battery performance^11^. To avoid contact loss on stripping, stack pressures on the order of megapascals are applied to cells, improving contact retention by creep and plastic flow^12^. However, high stack pressures increase the risk of electrolyte fracture and cell shorting^13^ and are impractical in a commercial vehicle.

Lithium-rich alloys are of interest to overcome these challenges^14^. Alloying modifies the physical properties of lithium metal such as the diffusion rate^15–17^, mechanics^18^, chemistry at the interface and the material microstructure; all of which can change the emergent electrochemical performance. This is achieved with only a small reduction of energy density compared to pure lithium^19^. Lithium-magnesium binary alloys have been considered one of the most promising alloy negative electrode candidates^14^ due to their high energy density, wide solid solubility in the lithium-rich phase and similarity in processing to lithium metal. Conflicting literature reports on diffusivity^20^ have contributed to this interest. Two works, using different methods, have identified similar decreases in lithium diffusivity with increasing magnesium alloy content^15,16^, but recently refs. ^16, 20^, investigating the electrochemical behaviour of lithium lanthanum zirconium oxide solid-state electrolyte, demonstrated improved stripping performance with increased magnesium content. This result shows that measurement of the diffusivity alone is insufficient to understand the behaviour of alloy negative electrodes. Diffusivity, mechanical properties, surface chemistry and microstructure all contribute to the electrochemical performance.

Here we investigate the link between materials properties and electrochemical performance in the lithium-magnesium alloy system in combination with argyrodite, Li_6_PS_5_Cl, sulphide solid electrolyte. We synthesise and microstructurally characterise alloys of three magnesium compositions; then measure the mechanical hardness, stiffness, adhesion and creep of these alloys by nanoindentation - quantifying the change with magnesium content. The effect of magnesium on the interfacial chemistry is explored with electrochemical impedance spectroscopy (EIS) and x-ray photoelectron spectroscopy (XPS). Diffusivity is measured using ^6^Li pulsed field gradient nuclear magnetic resonance spectroscopy (PFG-NMR). These properties are correlated with electrochemical tests of symmetric cells for varied magnesium content, performed in tightly controlled, realistic conditions (30 °C, 50–100 μm thick electrodes, 2.5–10 MPa stack pressure), with corresponding cross-sectional electron microscopy and a stress-coupled continuum lithium transport model used to understand the underlying processes more clearly. We show how magnesium aids contact retention on stripping but negatively affects lithium diffusivity and demonstrate how light magnesium alloying is an effective avenue to balance these two contrasting effects.

## Results and discussion

### Synthesis and characterisation

**Fig. 1:**
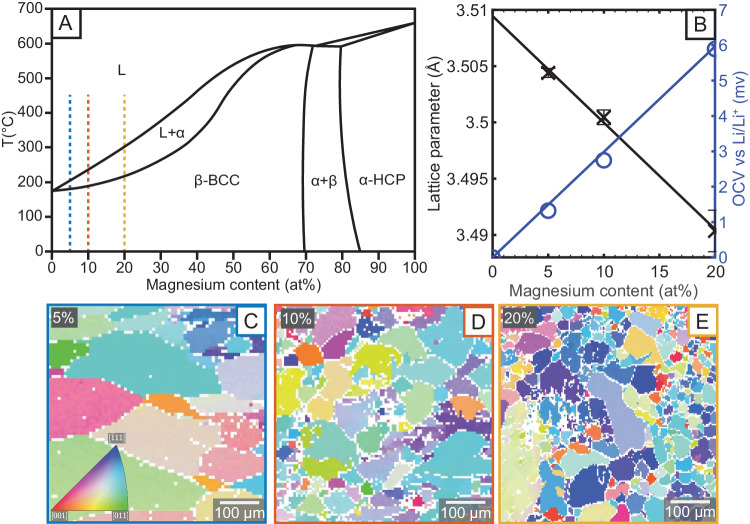
Physiochemical characterisation of the Li-Mg alloys. **A** Binary phase diagram of the lithium magnesium system, redrawn from ref. ^57^ with labelled phase regions of liquid (L), the lithium rich body centred cubic phase (*β*), the magnesium rich hexagonal close packed phase (*α*) and the two-phase region (*α* + *β*). The investigated compositions are marked with dotted lines in 5% blue, 10% orange, 20% yellow. **B** Lattice parameter measured by X-ray diffraction and open circuit voltage (OCV) measured against lithium metal. **C**–**E** Representative EBSD maps of 5, 10 and 20 atomic percent alloys respectively, outlined in the respective colours marked on the phase diagram.

Lithium magnesium alloys were prepared using a box furnace within an argon-filled glovebox (*O*_2_, *H*_2_*O* < 0.1 ppm). The synthesis was informed by the phase diagram, a schematic of which is presented in Fig. 1A. The alloys were characterised by X-ray diffraction (Supplementary Fig. 1 and Supplementary Note 1). The lattice parameters obtained are plotted in Fig. 1B, (*a* = 3.5095–0.0009*x *Å, where *x* is at% Mg) which closely matches previous work^21^, suggesting the alloy content matches their nominal content within 1%. Asymmetric solid-state cells with lithium metal counter electrodes were assembled for each alloy sample. The open circuit voltage (OCV) against lithium metal for each alloy composition is plotted in Fig. 1B, (*E* = 0.29*x mV* vs Li/Li^+^)^20,22^. Samples from each alloy were characterised in the scanning electron microscope (SEM). Energy dispersive x-ray spectroscopy (EDX) showed magnesium was evenly distributed throughout all alloy samples, and backscattered electron images were uniform (Supplementary Fig. 2). To characterise the underlying grain structure, the samples were mapped using electron backscattered diffraction (EBSD). This was achieved by using microtome blades to prepare flat surfaces^7^. Representative maps of as-cast samples, cooled from 450 ^∘^C outside of the furnace, are shown in Fig. 1C–E. The conventional colour scheme has been used with red corresponding to an < 001 > surface normal, green corresponding to < 110 > and blue to < 111 > as shown in the inverse pole figure inset in Fig. 1C. The mean grain diameters and standard deviations of the maps shown are 116.7 ± 65.2 μm, 50.6 ± 34.4 μm and 33.5 ± 21.6 μm for the 5, 10 and 20% alloys respectively.

The specific microstructure of the alloys depends strongly on the thermal history of the sample. However, even with the same thermal treatment, increasing magnesium content leads to the stabilisation of finer microstructure, with the average grain size decreasing. With increased magnesium content, and slower cooling rates, we observed a greater tendency for a dendritic microstructure, driven by constitutional super-cooling in solidification (Supplementary Fig. 3). This is seen in the EBSD as large regions with the same crystallographic orientation, but with sub-grain boundaries of low band contrast. A small region (light green orientation) can be observed in the bottom left corner of Fig. 1E.

### Mechanical properties

**Fig. 2:**
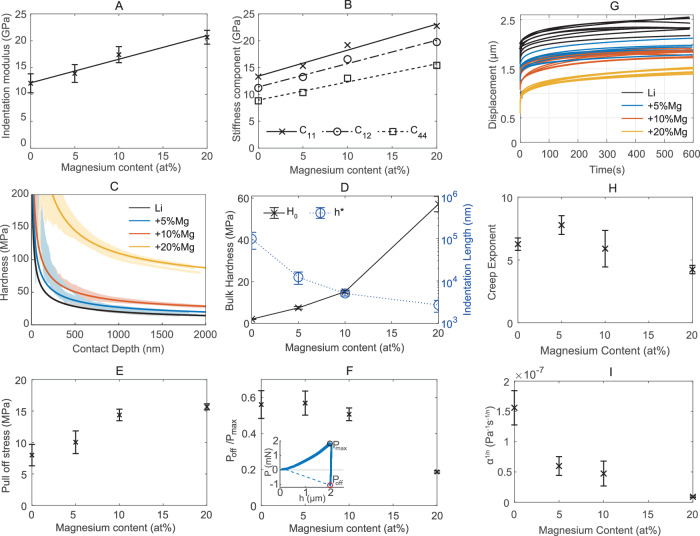
Elastic, plastic and viscoplastic mechanical properties of lithium magnesium alloys. **A** Average indentation modulus with magnesium content, error bars show standard deviation. **B** Elastic stiffness components with magnesium content. **C** Average fit size-dependent hardness curves for each alloy composition with representative data plotted in lighter colour. **D** Fitting parameters for curves shown in (**C**), showing bulk hardness (*H*_0_) and characteristic decay length (h^*^), error bars show standard deviation. **E** Pull-off stress with magnesium content, error bars show standard deviation. **F** Relative adhesion with alloy content, insert showing example load-displacement curve with maximum load (*P*_*max*_) and pull-off load (*P*_*off*_) marked, error bars show standard deviation. Pure lithium values are reproduced from an earlier work by the authors^7^. **G** Creep curves for 1000 μN of constant load for all alloy compositions. **H** Creep exponent, *n*, with magnesium content, error bars show standard deviation. **I** Creep prefactor, *α*, with alloy content, error bars show standard deviation.

Lithium alloy samples of 5, 10 and 20 at% magnesium were mechanically characterised using nanoindentation, repeating the methodology used for pure lithium metal^7^. Fresh flat surfaces of alloy ingot samples were prepared using a microtome blade. A series of indents around 1500 nm in depth were performed across grains with a range of crystallographic orientations to measure the modulus and hardness (Supplementary Fig. 4). In Fig. 2A, a linear increase in average indentation modulus was observed with increasing magnesium content (M = 0.44*x* + 12.2 GPa). This trend extrapolates to stiffness observed in magnesium-rich beta alloys^23^. The error bars on Fig. 2A correspond to the standard deviation of the data set, with different crystallographic orientations having different moduli. The EBSD coupled indentation method outlined in previous work^7,24^ was repeated to extract the three independent stiffness tensor components C_11_, C_12_ and C_44_ of each alloy, which are plotted in Fig. 2B and detailed in Table 1 with the Hill polycrystalline estimate values calculated. These results are in agreement with single crystal work investigating up to 4.2 at%Mg^18^. The elastic response to a general stress state can be calculated from these values. There is a clear linear increase in the three components, with minimal change in anisotropy.

**Table 1:** Elastic properties of lithium magnesium alloys measured by EBSD coupled indentation at 25  ^∘^C

Alloy	*C*_11_	*C*_12_	*C*_44_	B	G^a^	E^a^	ν^a^
Li	13.3	11.2	8.8	11.9	4.0	10.7	0.35
5%	15.3	13.2	10.3	13.9	4.4	12.0	0.36
10%	19.2	16.6	13.0	17.5	5.6	15.1	0.36
20%	22.8	19.7	15.4	20.7	6.6	17.8	0.36

Superscript^a^ denotes Hill polycrystalline estimate.

*B* bulk modulus, *G* shear modulus, *E* Young’s modulus, *ν* Poisson’s ratio.

Like pure lithium^25^, strong size-dependent hardness is observed for lithium magnesium alloys. The average fitting curve for all indents at each composition is plotted in Fig. 2C with a representative data set, and the average fitting parameters are plotted in Fig. 2D. The observed hardness with indentation depth was fitted with Eq. (1) taken from Nix and Gao’s work^26^, using data beyond 500 nm indent depth, such that the measured hardness decays towards *H*_0_ with a characteristic decay length h*. A large increase in bulk hardness *H*_0_ is observed, increasing from 2 MPa for pure lithium to over 57 MPa for the 20% alloy. This is reflected when handling the bulk ingots where the high-content alloys require considerably more force to deform.

1 $$H={H}_{0}\sqrt{1+\frac{{h}^{*}}{h}}$$

A corresponding decrease in the characteristic decay length is observed (Fig. 2D), following the expected trend relative to the bulk hardness^26^. The dislocation density in the lattice of a material is inversely proportional to the measured decay length^27,28^ meaning we expect the stored lattice dislocation density to be multiple times greater for the 5, 10 and 20% alloys, than for pure lithium. This may lead to less voiding, caused by the aggregation of vacancies introduced during stripping at the electrode∣solid electrolyte interface^3^, with a higher rate of excess vacancy absorption by dislocation climb. Improved contact retention has been observed by several works on the lithium-magnesium system^16,20,29^. On indenter tip retraction, significant adhesion was observed for all indents, with a similar magnitude of force required to un-stick the indenter tip from each alloy as was needed to indent the sample. The mean pull-off stress (retraction force/contact area) as a function of magnesium content is plotted in Fig. 2E. An example load-displacement graph is shown in the inset of Fig. 2F. There is a clear increase in pull-off stress with magnesium content. A numerical solution to calculate work of adhesion for Berkovich indentation with extensive plastic deformation is yet to be found^30^, but considering a purely elastic assumption given by the Kendall model (${P}_{off}=\sqrt{8{A}_{c}M{W}_{ad}R}$, $R=\sqrt{{A}_{c}/\pi }$), gives work of adhesion values of 9.61 ± 0.03 mJ m^−2^ for pure lithium and 15.00 ± 0.57, 26.71 ± 0.56 and 24.45 ± 0.34 mJ m^−2^ for the 5, 10 and 20 % alloys respectively. Recent works have suggested higher electrode-electrolyte adhesion leads to lower stack pressures being needed to prevent interfacial voiding on electrochemical stripping^31^. The ratio of peak indent load (P_*max*_) and the retractive load required to overcome tip adhesion (P_*off*_) is plotted in Fig. 2F. There is a substantial decrease in this relative strength of adhesion with higher magnesium content. This is a result of the increased hardness with higher magnesium content. This lower effective adhesion makes the alloys easier to work with; it can be rolled thinner before rolling defects are observed, and it does not adhere to tweezers when handled.

Indentation creep tests were performed, maintaining a constant load of 1000 μN with a Berkovich tip for 600 s, following the methodology outlined in other works^32^. Creep curves for pure lithium and the three alloys are plotted in Fig. 2G and follow the expected trend, with less creep for higher magnesium content. The methodology of^33^ was followed to calculate equivalent uniaxial creep parameters from indentation creep data. The creep exponent, n, for pure lithium, was measured to be 6.26 with a standard deviation of 0.497. This is in line with the bulk uniaxial test values for lithium of 6.4^34^, 6.56^12^ and 6.6 ± 0.7^35^. Given the large exponent, and the stresses that drive creep being of the order of MPa, the prefactor *α* varies significantly depending on the measured creep exponent. We found the value *α*^1/*n*^ far more consistent between indents. For pure lithium *α*^1/*n*^ = 1.56 × 10^−7^*Pa*^−1^*s*^−1/*n*^, with a standard deviation of 2.8 × 10^−8^. Using these values the prefactor for lithium can be calculated as 2.29 × 10^−43^*Pa*^−*n*^*s*^−1^. This is in agreement with the literature value of 2.95 × 10^−43^*Pa*^−*n*^*s*^−1^ at 30 ^∘^C^35^. This agreement validates the indentation methodology and suggests that the mechanisms of creep at the microscopic and macroscopic scales are the same. The fit values for the lithium magnesium alloy samples are presented in Fig. 2H, I and in Supplementary Table 1. The creep exponent is in the range of 3–10 for all alloys suggesting the mechanism is dislocation glide. It remains approximately constant up to a magnesium content of 10%, and then is lower for the 20% sample. The prefactor significantly decreases with higher magnesium content. This follows from the hardness data which shows dislocation glide is substantially harder with more magnesium present.

The substantial increase in creep-resistance is a consequence of the increased hardness (Fig. 2D) and decreased thermal activity of the material with higher magnesium content, with the homologous temperature decreasing from 0.69 for pure Li to below 0.6 for the 20% magnesium alloy. The measured prefactors and exponents mean that higher magnesium content alloys, under the same stress, will have orders of magnitude lower creep rates than lower content alloys.

### Interfacial chemistry

Pure magnesium metal is known to have a native passivation layer, which limits its use in batteries, it is therefore critical to quantify the impact of magnesium alloying on the interfacial chemistry. Lithium alloys have different lithium activity and potential compared to pure lithium leading to changes in the charge transfer resistance, passivation layer composition and, possibly, the chemistry of the SEI formed, leading to changes in interfacial impedance^10^. By performing EIS at regular intervals throughout an open circuit stabilisation period on multiple self-symmetric cells with an argyrodite solid electrolyte, we have quantified the interfacial resistance for the electrode-electrolyte interface for each alloy, and its growth rate.

**Fig. 3:**
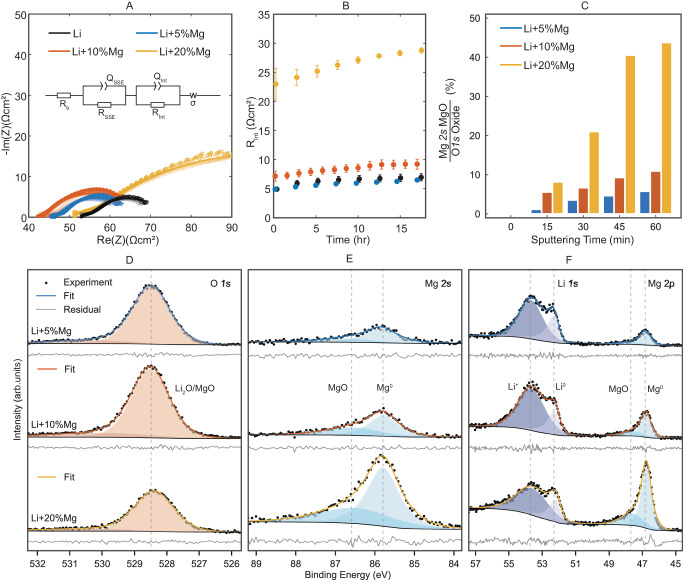
Electrochemical and physiochemical characterisation at the electrode∣electrolyte interface. **A** Electrochemical impedance spectroscopy measurements and data fit of self-symmetric cells over 16 h at 30 ^∘^C, 5 MPa stack pressure. Inset: equivalent circuit used to fit the raw EIS data. EIS data and fits are available in the repository^56^. **B** Interfacial resistance against time. Error bars show standard error across multiple samples. **C** Bar chart showing magnesium oxide fraction of total oxide at a series of sputtering depths. **D**–**F** Ex situ XPS measurements and analysis of the passivation layers on alloy samples. Core-level XPS spectra for each alloy composition for (**D**) O *1s*, (**E**) Mg *2s*, (**F**) Li *1s* and Mg *2p*.

Representative examples of impedance spectra for each alloy are plotted in Fig. 3A. The spectra are fit with the equivalent circuit inset in Fig. 3A^36^. The small high-frequency component, attributed to grain boundaries or porosity within the solid electrolyte^37^, was effectively constant throughout the open circuit stabilisation period and there is no significant trend of this or ohmic resistance with magnesium content. The Warburg element, describing transport within the electrode material^38^, was fixed based on diffusivity values calculated from ^6^Li PFG-NMR reported later. As has been discussed in the literature^37^ the large, low-frequency component corresponds to the combined processes of charge transfer and transport through the SEI and passivation layers. It was not possible to robustly separate these two processes, so they are grouped together as a single total interfacial resistance^39^.

The extracted areal interfacial resistance (*R*_*Int*_) per electrode is plotted against time in Fig. 3B, with error bars indicating the standard error across the samples measured. The pure lithium and 5% Mg samples are indistinguishable, but the 10% and 20% Mg have a larger interfacial resistance which grows at a proportionally higher rate with time.

Charge transfer resistance is a function of the activity of lithium in both the solid electrolyte and the alloy^40^. This is unlikely to account for the size of change in interfacial resistance observed and should remain constant over time. Considering transport through the SEI, the change in potential with magnesium alloying is small (Fig. 1B) and therefore, we would expect near-identical electrochemical decomposition products of the solid electrolyte. XPS results of magnesium sputtered in situ onto the electrolyte show that it is challenging to distinguish any magnesium compounds formed from the lithium compounds that we know will be present (Supplementary Fig. 5 and Supplementary Note 2). Of the possible decomposition products, all of the lithium compounds (Li_2_S, LiCl, LiOH) are more thermodynamically stable than their magnesium equivalents (MgS, MgCl_2_, Mg(OH)_2_), with the exception that magnesium oxide ($\Delta{\rm{G}}_{MgO}^{f,303K}$= −607.8 kJ mol^−1^) is more stable than lithium oxide ($\Delta{\rm{G}}_{L{i}_{2}O}^{f,303K}$= −573.6 kJ mol^−1^). The increased interfacial resistance and increased growth over time therefore suggests that there is a change in the composition of the passivation layer on the electrodes due to the presence of magnesium in the electrode.

Studies on the lithium metal passivation layer indicate a bi-layered structure, a lithium oxide-rich region in contact with lithium metal, with a surface layer containing primarily lithium carbonate and hydroxide^41^. Based on the similar, negative formation enthalpies of lithium and magnesium oxide, the composition of the oxide layer is likely to change in the alloys. To investigate this, XPS was performed on the alloy samples, prepared in the same way as the cell electrodes. Figure 3C plots the relative intensity of the magnesium *2s* peak and total oxide peak at a range of sputtering times. As can be seen, there is some variation in relative composition over the thickness of the layer, with more magnesium compounds present deeper into the layer and more lithium compounds at the surface, in agreement with the study on pure lithium^41^. Full spectra at different sputtering times can be found in Supplementary Fig. 6. Figure 3D–F show the spectra for O *1s*, Mg *2s*, Li *1s* and Mg *2p* for the three alloy compositions after 60 min of sputtering. Given the thermodynamics discussed, we attribute the Mg *2s* peak (B.E. ~ 86.7 eV) to magnesium oxide. The lithium *1s* peak (B.E. ~ 53.7 eV) is a combination of several compounds including lithium carbide^42,43^, oxide, hydroxide and others. All three alloy passivation layers consist of a combination of magnesium oxide and lithium compounds, but the relative fractions vary. It seems with a higher magnesium alloy content, a much greater fraction of magnesium oxide is observed in the sputtered passivation layer. Given magnesium and lithium oxides do not form a solid solution, we expect magnesium oxide to inhibit lithium transport. The XPS observations, therefore, correlate well with the increased interfacial impedance observed by EIS.

### Diffusivity

**Fig. 4:**
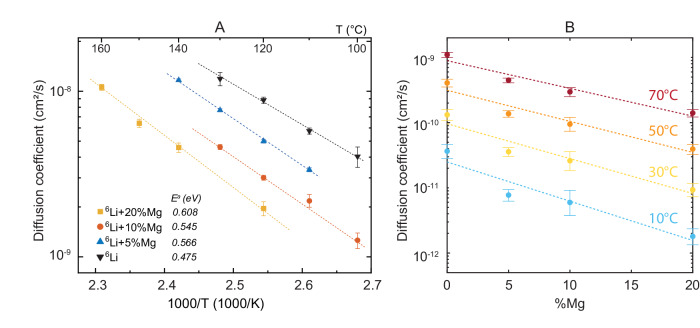
Diffusivity measurement of 0, 5 and 10 and 20% Mg lithium alloys. **A** ^6^Li diffusion coefficient of pure lithium and LiMg alloy samples measured by PFG-NMR. **B** Calculated diffusion coefficient as a function of temperature and magnesium content. Error bars show standard error.

Self-diffusivity of lithium within the alloys was measured using PFG-NMR^44^. The measured values are shown in Fig. 4A and Supplementary Table 2. By fitting the Arrhenius expression to the measured data, values can be calculated for a range of reasonable operating temperatures (Fig. 4B). This measurement is challenging in naturally isotopic lithium due to the combination of slow ionic diffusion and fast ^7^Li spin relaxation caused by a large quadrupolar interaction^45^. To measure diffusivity in the magnesium alloy samples, ^6^Li enriched alloy samples were synthesised using the same procedure as the non-isotopic alloys. The ^6^Li isotope has a much smaller nuclear electric quadrupole moment and therefore a longer relaxation time^46^. High temperatures were used to increase the diffusion rate to measurable values. Fitting the diffusion coefficients at different temperatures with the Arrhenius equation gives an activation energy of 0.475 ± 0.020 eV, which fits previous literature reports well^47^. Lower values of diffusivity and higher activation energies are measured for increasing magnesium content, with measured values of 0.566 ± 0.015, 0.545 ± 0.036 and 0.608 ± 0.020 eV for the 5, 10 and 20% samples respectively, close to the previously reported value 0.63 eV for the Li + 10%Mg alloy measured with the motional narrowing of ^7^Li peaks^48^. The activation energy has two components, one due to the enthalpy of vacancy creation and the second the enthalpy of vacancy hopping. An increase in vacancy creation enthalpy is expected with a higher melting point. The increase in hopping enthalpy with increasing magnesium content is attributable to closer lattice packing and increased stiffness. There is some experimental scatter on the activation energy values, but the relationship appears linear (E_*a* _= 0.4993 + 0.0057*x* eV, where *x* is at%Mg). The ^6^Li diffusion coefficient of pure lithium and Li + 20%Mg at room temperature (298 K) is calculated to be 9.73 × 10^−11^ and 6.31 × 10^−12^ cm^2^ s^−1^ by the extension of the Arrhenius plots, close to reported ^8^Li NMR results^15^ and recent time-of-flight secondary ion mass spectrometry results^16^. Electrochemically measured diffusivity of order 10^−8^ cm^2^ s^−1^ at room temperature^17^ is an outlier.

### Stripping performance

**Fig. 5:**
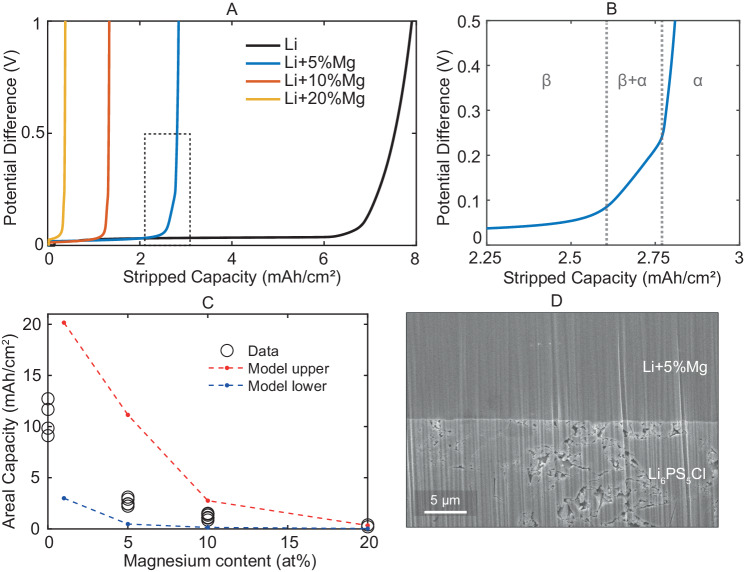
Electrochemical and physiochemical characterisations of symmetric Li-Mg∣∣Li-Mg cells. **A** Chronopotentiometric stripping showing potential as a function of areal capacity at 0.31 mA cm^−2^, 30 ^∘^C, 5 MPa. **B** Zoom from A on 5% Mg curve potential increase region. **C** Extracted capacity before 5 V limit for self-symmetric cells of varying alloy content. Model predictions for constant diffusivity in red and variable diffusivity in blue. **D** Ex situ cross-sectional SEM image of Li+5at%Mg - Li_6_PS_5_Cl interface after chronopotentiometry, prepared by plasma focused ion beam milling.

To investigate stripping performance, self-symmetric cells for each alloy composition were assembled and stripped in one direction at a constant current density. The conditions were chosen to replicate more realistic cell operating discharge conditions with a constant stack pressure (5 MPa) applied with a spring, room temperature (30 ^∘^C) and a low current density (0.31 mA cm^−2^) in one direction^49^. Representative potential-capacity curves are plotted in Fig. 5A. There is a clear trend of reduced accessible capacity with increasing magnesium content. As can be seen in Fig. 5A and B, the shape of the cell potential increase in the alloys is different to the case of lithium failure. The zoomed Fig. 5B shows there are three distinct stages of potential increase. The first and longest is a gradual increase. This corresponds to the increasing magnesium content of the alloy at the surface as the concentration gradient builds. This stage ends when a potential difference of ~70 mV is measured, this transition point corresponds to the saturation limit of the body-centred cubic (BCC) alloy phase (69 at% Mg)^22^. The second stage is approximately linear, with a steeper gradient. After ~30 min, a third stage of rapid potential increase towards very large potentials is observed. The latter two stages correspond well to the two-phase and alpha-phase regions of the lithium magnesium phase diagram. The diffusivity of lithium in the alpha phase is orders of magnitude slower than in the beta phase. The diffusivity of lithium in pure *α*-Mg at 30 ^∘^C is reported to be 7 × 10^−22^ cm^2^ s^−1^^[ 50^, therefore as the lithium-depleted surface layer transforms from BCC beta phase to hexagonal close-packed alpha phase there is an increase in the concentration gradient required at the interface to supply the fixed current density (Fick’s first law), and therefore a substantial increase in the transport overpotential within the negative electrode. This leads to a rapid increase in measured potential difference. Pure lithium only has one component and therefore no phase transformations, its increase in potential is smooth.

The total capacity extracted for each cell is plotted for each alloy content in Fig. 5C, showing good reproducibility for all three alloy compositions and pure lithium. It is essential to do multiple tests to capture the inherent variability (Supplementary Fig. 7).To investigate the impact of changes in materials properties with alloying on stripping performance, we have developed a poro-viscous theory for lithium transport coupled to creep in Li-Mg alloys ((Supplementary Figs. 8–10 and Supplementary Note 3)), building on the continuum theory presented in ref. ^51^. According to the model, the stripping of lithium near the interface induces volume contraction of the host alloy, which must be accommodated by creep due to lateral geometric constraints. Creep in turn requires the generation of a transient, heterogeneous stress field. Diffusion-induced stresses contribute to the driving force for lithium transport, accelerating diffusion in the direction of increasing hydrostatic tension. The model assumes perfect interfacial contact and therefore only describes transport, not contact retention. The model was implemented in an in-house finite element code with fully implicit time integration. Physically we believe this accurately reflects the reality, with cross-sectional SEM characterisation for samples prepared by focused ion beam showing a dense electrode morphology after the chronopotentiometric stripping Fig. 5D, this is unsurprising given the material’s low hardness and substantial creep.

We used the model to calculate the stripping capacity for the prescribed stripping current (0.31 mA cm^−2^), temperature 30 ^∘^C and stack pressure (5 MPa). Creep parameters were directly taken from our indentation creep measurements. The diffusion coefficient of lithium in the alloys was estimated by extrapolating data in Fig. 4 to 30 ^∘^C using the Arrhenius relation Fig. 4B. The values used are available in Supplementary Table 3. In the simulations, diffusion coefficients were either assumed constant and set to their extrapolated value at 303 K for the initial alloy composition, or taken as a function of the local alloy composition as it evolves during stripping. The predicted total capacity extracted for each alloy content is plotted alongside experimental data in Fig. 5C. There is a clear correlation between the lithium diffusivity and the total stripping capacity, with predicted capacity decreasing as the diffusion coefficient of lithium in the alloy decreases. Simulations where the diffusion coefficient is constant overestimate the experimental capacity, while simulations where the diffusion coefficient is a function of the local composition underestimate the experimental capacity. The fact that the experimental capacity sits somewhere between these two limits suggests that the diffusion coefficient of lithium in the alloy during stripping does not decrease as much as predicted by a simple regression, which could be attributed to a higher vacancy concentration than the equilibrium concentration for the local composition during stripping.

A key prediction of the model is that extracted capacity is almost independent of stack pressure. According to the model, increasing the stack pressure increases the chemical potential of lithium in the alloy (favouring lower lithium concentration at equilibrium), but does not impact the driving force for lithium transport, since the gradient of the stack pressure is zero. The model does predict the development of hydrostatic stress gradient in the alloy associated with the stripping of lithium, whose magnitude is governed by the creep model, however the effect of stress gradient on the transport kinetics remains negligible compared to the effect of composition gradient. In short, to optimise transport, low magnesium content is desirable.

**Fig. 6:**
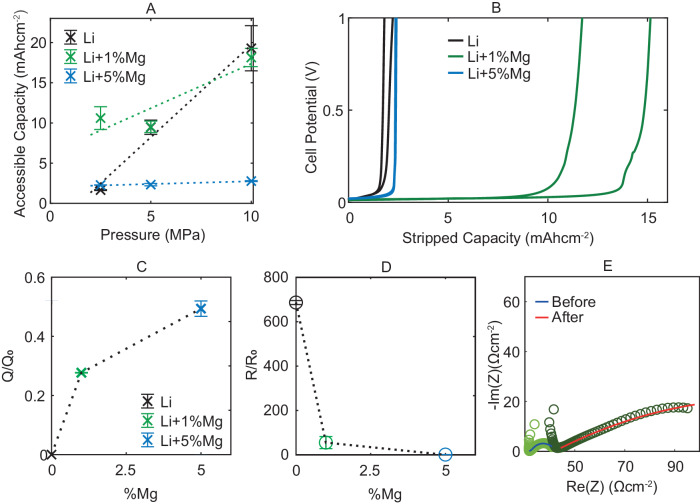
Electrochemical characterisation of symmetric 0, 1 and 5% Mg Li-Mg∣∣Li-Mg cells at varied pressure. **A** Areal capacity extracted before a cell potential of 100 mV is reached at a range of pressures, error bars show standard error across multiple samples. **B** Unidirectional chronopotentiometry of self-symmetric cells at 2.5 MPa. **C** Ratio of interfacial capacitance before (*Q*_0_) and after (Q) unidirectional chronopotentiometry, error bars show standard error across multiple samples. EIS data and fits are available in the repository^56^. **D** Ratio of interfacial resistance before (*R*_0_) and after (R) unidirectional chronopotentiometry, error bars show standard error across multiple samples. **E** representative EIS spectra before/after chronopotentiometry for self-symmetric 1% Mg alloy cell at 2.5 MPa fit with equivalent circuit inset in Fig. 3A.

Experimentally, stack pressure was varied between 2.5 and 10 MPa for chronopotentiometry at the same current density of 0.31 mA cm^−2^ to a cut-off voltage of 5 volts at a temperature of 30 ^∘^C. As before, the potential was low before rapidly increasing. Figure 6A shows the areal capacity extracted before a cell potential of 0.1 V is reached.

Comparing the pure lithium and 5% Mg alloy, clear differences can be seen. For the 5%Mg alloy, the extracted capacity is essentially constant across the pressure range, confirming the prediction of the model. Pure lithium’s accessible capacity, by comparison, is highly dependent on stack pressure, with below 2 mAh cm^−2^ extracted at 2.5 MPa, compared to over 15 mAh cm^−2^ at 10 MPa. There is a dynamic process of contact retention which stack pressure aids. Crucially at a low stack pressure of 2.5 MPa (Fig. 6B), despite a much lower diffusivity, the 5% Mg alloy outperforms pure lithium.

Impedance spectroscopy performed before chronopotentiometry and 1 h after the 5 V limit was reached can be fit, as described earlier, to obtain an interfacial equivalent capacitance and resistance. The ratios of the before and after values for cells with 2.5 MPa of stack pressure are plotted in Fig. 6C and D. For pure lithium, the interfacial equivalent capacitance (Q) goes to approximately zero and the interfacial resistance (R) increases around 700-fold, indicating a near-total loss of contact with the solid electrolyte. By comparison, the 5% Mg interfacial equivalent capacitance decreases by 50% and the interfacial resistance remains approximately constant; suggesting much greater interfacial contact retention - with the cell potential increasing due to the diffusivity limit of the alpha phase as discussed earlier.

A balance exists between fundamental transport kinetics in the material, which are slower with a higher magnesium content and contact retention at the interface, which improves with a higher magnesium content. A 1% Mg alloy was synthesised and tested, with the results plotted in green in Fig. 6. Particularly at 2.5 MPa of stack pressure, the 1% Mg sample delivers significantly greater capacity than both pure lithium and the 5% Mg alloy (Fig. 6B). The 1% alloy combines the fast diffusion kinetics of pure lithium with much of the contact retention and stress independence of the 5% magnesium alloy. Figure 6C shows after the chronopotentiometry test, the interfacial capacitance decreased to 28% of its initial value, indicating significant contact retention; although clearly worse than the 5% Mg alloy cells. Representative 1% Mg EIS spectra before and after the test are inset in Fig. 6E.

When operating in low stack pressure conditions, the addition of small amounts of magnesium aids in contact retention with the solid electrolyte; offsetting the diffusivity penalty. It is surprising that just 1% magnesium addition significantly aids contact retention compared to pure lithium, enabling much larger capacities to be extracted. On any successive charge, the increased contact area reduces the risk of current concentrations and associated cell shorting^3^. A compromise exists between accessible capacity, contact retention and the temperature required for successful operation.

Alloying lithium with magnesium benefits contact retention at the interface with the solid-state electrolyte on stripping, especially at the low stack pressures which are practical in a large battery. This effect must be balanced against the decrease in lithium transport kinetics due to magnesium alloying. The wide solid solubility window of the magnesium-lithium system allows a large capacity to be withdrawn from the alloy before a detrimental phase transformation occurs.

In addition to the benefits to electrochemical performance, magnesium alloying changes the microstructural, mechanical, kinetic and chemical properties of lithium. Higher hardness of the magnesium alloys makes the thin calendared foils more durable and easier to work with, aiding manufacturing. The fundamental mechanical and diffusivity values measured in this work enabled a stress-coupled model of lithium transport to be developed and the electrochemical behaviour to be understood. EIS measurements shows only a small increase in interfacial impedance for the alloys compared to pure lithium. The alloys have nearly identical operating potential, therefore the electrolyte reduction layer is likely to be similar. Differences are attributed primarily to changes in the alloy passivation layer composition. A consistent theme we observed throughout this work, is that even minimal magnesium alloying makes many characterisation methods easier - increased atomic number leads to improved EBSD resolution, XRD signal and stability in PFIB. Finally, at practical stack pressures (<5 MPa), light alloying of lithium (<5%) with magnesium leads to improved electrochemical performance compared to pure lithium metal.

## Methods

### Synthesis

Lithium metal (foil, 99.9%, Alfa Aesar) and magnesium metal (turnings, 99.98%, Alfa Aesar) were used for the casting of the Li-Mg alloys in a box furnace (MTI KSL-1200X-J-UL) within an argon-filled MBraun glovebox (O_2_ and H_2_O < 0.1 ppm). Lithium metal (~1 g) was heated to 450 ^∘^C, well above the Li-Mg liquidus temperature, in a custom-made stainless-steel crucible, lined with molybdenum foil (Goodfellow), with a molybdenum foil lid. The molten lithium was removed from the furnace once at temperature, any slag carefully removed with a stainless-steel spatula and an appropriate mass of magnesium turnings were fully dissolved in the molten lithium using a stainless-steel spatula to stir. The crucible was then returned to the furnace for 2 h at the same temperature before slowly furnace cooling to room temperature. The foil liner was easily removed from the ingot with pliers. Smaller volumes (0.1–1 cm^3^) for experiments were cut from the bulk ingot using microtome blades (Thermo Scientific Epredia™ Shandon™ Premium High-profile Disposable Blades).

### Electron microscopy

SEM, EBSD mapping, electron dispersive spectroscopy (EDX) and nanoindentation were performed within a single contained system consisting of a TESCAN Mira3 FEG-SEM with Oxford Instruments EBSD and EDX detectors, opening into a custom MBraun glovebox. EBSD was performed at 70 degrees tilt, a beam voltage of 20 kV and a current of 500 nA. The quality of the EBSD pattern and achievable resolution improved greatly with magnesium content, due to the increased electron cross-section of the material.

### Cross-sectional PFIB

After stripping, the cells were disassembled and placed on stubs for microscopy characterisation. The samples were stored under Ar in a glovebox (H_2_0 and O_2_ < 0.1 ppm) until they could be transferred to the microscope using an airless transfer vessel (Gatan iLoad) to the plasma FIB-SEM (Helios G4 PFIB CXe DualBeam, Thermo Scientific) for characterization. All samples were sectioned using the same milling and polishing procedure, final ion beam polishing parameters of 30 kV and 15 nA were used as a trade-off between maximising milling rate and minimising beam damage. The alloys were milled at room temperature as they had been confirmed to not suffer excessive artefact creation at these temperatures, however, lithium samples were milled at cryogenic temperatures (−150 ^∘^C) using a cryogenic stage (Gatan) as lithium’s low melting point leads to excessive image artefacts if milled at room temperature.

### Indentation

Small alloy samples (~3 mm × 3 mm × 1 mm) were mounted on custom 5 mm aluminium pin stubs with two-part Araldite epoxy. Indentation was performed using a Bruker Hysitron PI88 in situ nanoindenter, with a five-axis sample stage mounted to the five-axis stage of the electron microscope. The SEM opens into an argon glovebox enabling airless transfer. The multi-axis stage configuration allowed for the sample to be rotated between EBSD and indentation positions; to allow the manual placement of indents at least 3 indent widths apart, at sites with specific grain orientations. Only large grains with good EBSD band contrast were tested, any indents accidentally placed on or near grain boundaries were excluded from analysis. Ten and twenty percent alloys were annealed for 24 h at 150 ^∘^C to increase grain size sufficiently for this experiment. All indents were performed at a frequency of 70 Hz. The diamond Berkovich indentation tip (Hysitron), which was scanned by atomic force microscopy (Bruker Dimension Icon) to extract a cross-sectional area function used in the calculation of reduced modulus and hardness from the measured stiffness, displacement and load. The tip was cleaned before each test under an optical microscope. Machine compliance was measured to be 8.02 nm/mN. Prior to each test, thermal drift rate was measured in contact, with preload of 10 μN, over 40 s. Indents were performed in Bruker’s open-loop nanoDynamic mode, with the load increased linearly over 30 s to a target load of 1500, 2500, 4000 and 8000 μN for the pure Li, 5%, 10% and 20% samples respectively to achieve similar indent depths. Both modulus and hardness values were obtained using the continuous stiffness measurement technique at a frequency of 70 Hz. Indentation modulus values were obtained by averaging the stiffness measured between 1000 nm and 1500 nm of depth—from which the indentation modulus (M) was calculated^24,52^ (*ϵ* = 0.75). The measured moduli of each of the indents within a grain were averaged to give a mean value of indentation modulus for each grain orientation. Stiffness tensor components were extracted with the methodology outlined in a prior work^7^, based on the work of Vlassak and Nix^53^, details are provided in Supplementary Note 4. Indentation creep experiments were performed using the same diamond Berkovich tip with a constant load of 1000 μN for 600 s, at a measurement frequency of 70 Hz for all samples. To calculate the contact area from the measured stiffness, the mean value of indentation modulus for each alloy was used. The measured creep exponent (*n*) is independent of this value. The displacement, calculated from changes in stiffness was plotted on a log-log plot with time with the gradient giving the creep exponent. Bulk creep pre-factors were calculated from fitted indentation pre-factors using the polynomial conversion equations outlined in ref.^33^ for 70^∘^ tip angle.

### X-ray diffraction

XRD was performed using a Rigaku Miniflex XRD contained within a glovebox. To minimise height error, <100 μm slices were cut from the as-cast alloy ingots using a microtome blade and mounted on low background silicon holders with petroleum jelly. Plotted lattice parameters were obtained by plotting the calculated lattice spacing obtained by fitting two Gaussian peaks (K_*α*_ and K_*β*_) to each major peak to calculate the lattice parameter, before plotting this against (cos^2^*θ*/*sinθ*) for each peak and extrapolating the trend to zero (large *θ*).

### ^6^Li PFG-NMR

The alloy samples for NMR tests were prepared with ^6^Li-enriched lithium metal (95% ^6^Li, 5% ^7^Li Chunks, Sigma Aldrich). All the metal bulk were cut into small pieces and packed in NMR tubes sealed by J-Young’s caps. The ^6^Li PFG-NMR experiments were carried out on a Bruker AVANCE III HD spectrometer with a magnetic field of 9.4 T (Larmor frequency of ^6^Li is 58.89 MHz) equipped with a 5 mm single-axis diffusion probe. A stimulated echo pulse sequence was applied with an effective gradient pulse duration (*δ*) of 2 ms, a diffusion time (Δ) between 300 and 800 ms and gradient amplitudes varying between 0.02 and 25 T m^−1^. A pulse delay of 1 s and a scan number of 128-512 were applied for each test.

### XPS

X-ray photoemission spectroscopy measurements were made using a PHI Versaprobe III instrument capable of generating focused, monochromatic Al K*α* X-rays at 1486.6 eV, under ultrahigh vacuum (chamber pressure ~10^−7^–10^−6^ Pa) conditions. Alloy samples were prepared within a glovebox in the same way as electrodes by brushing a fresh surface and then calendering. These were then transferred into the XPS chamber using a vacuum transfer vessel (ULVAC PHI GmbH) to avoid contamination and ambient exposure. A 500 μm × 500 μm area from each sample was analysed. XPS data from the surface were collected with the monochromator operating at a power of 25 W and an electron beam voltage of 15 kV. In-built electron and low energy Ar sources were utilised for charge neutralisation. A pass energy of 224 eV was used for wide-sweep survey scans while high resolution core-level spectra were collected using a pass energy of 55 eV. The passivation layer depth-profiling was achieved with consecutive XPS analysis and Ar+ sputtering (4 keV, 3 mm × 3 mm) for a total of 60 min. Acquired spectra were fitted, after the application of a Shirley background, with Gaussian-Lorenzian line shapes, except for lithium metal which was fitted with an asymmetric Voigt line shape, using CasaXPS software^54^. All spectra from alloy samples were charge referenced to the adventitious C *1s* peak or the Li_2_C_2_ peak at 285 and 282.4 eV^43^, respectively. Fitted regions were quantified and relative fractions of components were estimated using the relative sensitivity factors provided by CasaXPS.

For in-situ sputtering of Mg, an argyrodite sample pellet was mounted on a deposition sample holder, supplied by the manufacturer PHI^36^. A Mg foil (~500–700 μm thick), prepared by pressing Mg flakes (Sigma Aldrich, purity 99.9%) in a pellet die at 500 MPa for 5 min using a hydraulic press, was then mounted on to the vertical plate. The in situ sputtering of Mg was performed using an Ar+ ion gun at an ion accelerating voltage of 4 kV, with the beam being rastered over a 3 mm × 3 mm area. Photoemission spectra were taken with neutralisers switched off. The spectra collected during in situ sputtering were all charge referenced to Cl *2p*_3/2_ at 198.5 eV, considering that the position of the spectra remained unchanged throughout the sputtering process^37,55^.

### Electrochemical measurements

Lithium stress relaxes when held under compression at a constant displacement. Given lithium is stiff (E = 10.4 GPa), the strains associated with typical stack pressures are well below 1%, a fixed displacement cell setup cannot give reliable and fixed stack pressures. Therefore, to achieve fixed and reliable stack pressure for electrochemical tests, it is essential to have a compressed spring in mechanical series with the cell. All electrochemical measurements were performed within an environmental chamber at a temperature of 30 ^∘^C. Li_6_PS_5_Cl (Ampcera 10 μm) pellets were pressed thickness of 700 μm within custom Macor ceramic cell cylinders (diameter = 10 mm) with stainless steel plungers densified at a pressure of 370 MPa for 300 s. Each cast alloy was calendared down to a thickness of 50–150 μm before 10 mm diameter electrodes were hole punched and attached to the solid electrolyte at a pressure of 80 MPa. An excess of lithium or alloy was always used. When assembled, the cell has a compressed spring that maintains a constant stack pressure of 2.5, 5 or 10 MPa throughout cycling, with the pressure set using stainless steel spacers. To measure the OCV with alloy content, asymmetric Li-Mg∣∣Li cells were assembled, with a stack pressure of 5 MPa, with the mean cell potential measured over 24 h reported. Self-symmetric cells were assembled for each alloy. These cells were rested for 18 h (potentiostatic EIS performed approximately every 2 h) before the chronopotentiometric Li-stripping test at 0.31 mA cm^−2^ and a cut-off voltage of 5 V. EIS was performed potentiostatically in a frequency range of 10 kHz–10 mHz with 10 points per decade and 10 mV amplitude around the open circuit potential. PEIS was performed after 1 h at open circuit following chronopotentiometry.

## Supplementary information

Peer Review File

Supplemetary Information

## Data Availability

The data generated in this study have been deposited^56^ in the Figshare database 10.6084/m9.figshare.25472536.
