# Supplementary material for: The impact of magnesium content on lithium-magnesium alloy electrode performance with argyrodite solid electrolyte
Source: Nat Commun. 2024 May 27;15:4511. doi: 10.1038/s41467-024-48071-0 (PMC11519574; doi:10.1038/s41467-024-48071-0)
Supplement: Supplementary file 2 — Supplemetary Information [file 41467_2024_48071_MOESM2_ESM.pdf]

Supplementary Information

The impact of magnesium content on lithium-magnesium alloy electrode performance with argyrodite solid electrolyte.

Jack Aspinall[a, b], Krishnakanth Sada[a,b], Hua Guo[a,b], Souhardh Kotakadi[a], Sudarshan Narayanan[a, c], Yvonne Chart[a,b], Ben Jagger[a], Emily Milan[a], Laurence Brassart[d], David Armstrong[a,b], and Mauro Pasta[a,b,*]

[a]Department of Materials, University of Oxford, Parks Road, Oxford OX1 3PH, United Kingdom
[b]The Faraday Institution, Harwell Campus, Quad One, Becquerel Avenue, Didcot OX11 0RA, United Kingdom
[c]Department of Sustainable Energy Engineering, Indian Institute of Technology, Kanpur, 208016, India
[d]Department of Engineering Science, University of Oxford, Parks Road, Oxford OX1 3PJ, United Kingdom

[*]Corresponding author: mauro.pasta@materials.ox.ac.uk

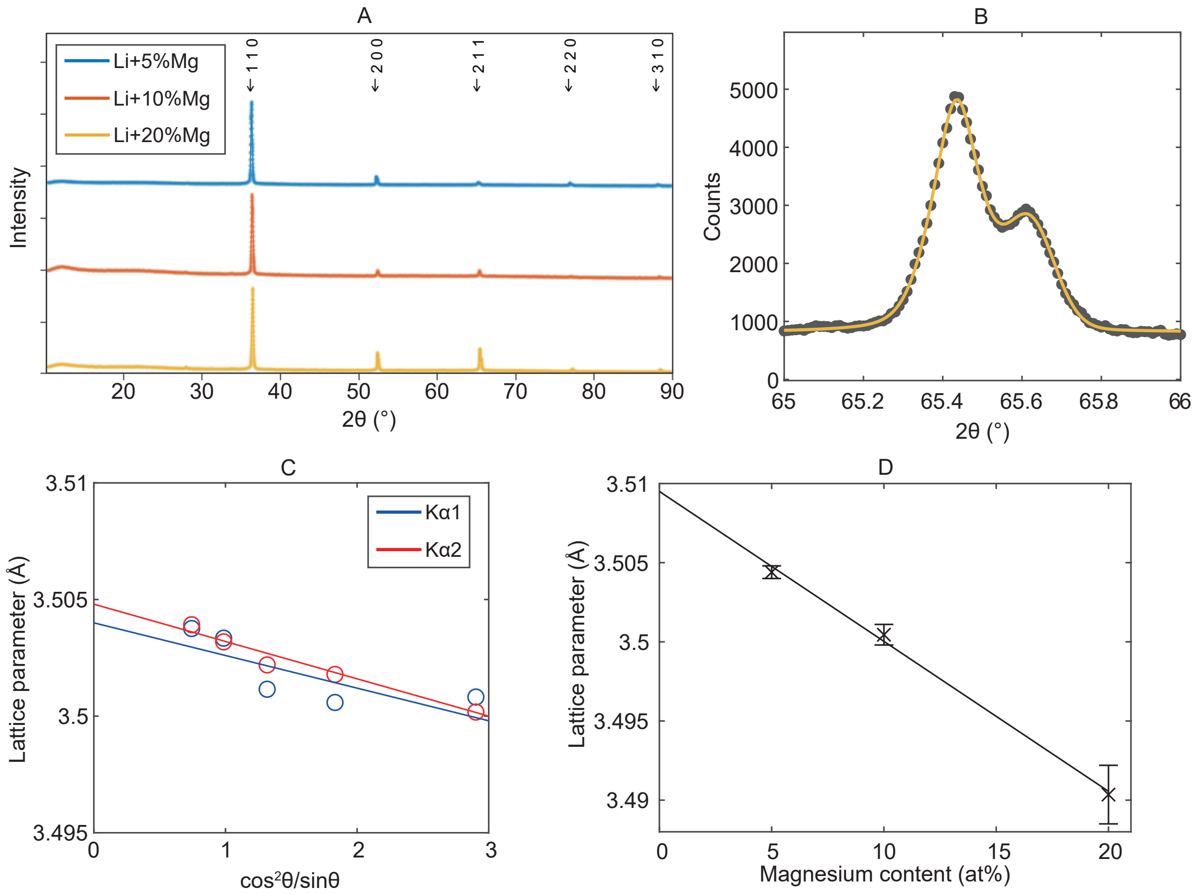

Supplementary Figure 1. XRD characterisation of lithium alloys. (a) Full XRD spectra of lithium alloys - y axes scaled for comparison. (b) Fitted peak example, 65 degree, Li+20%Mg (c) Extrapolation to theta = 0 for Kα1 and Kα2 peaks of Li+5at%Mg diffraction pattern. (d) Measured lattice parameters with magnesium content. Trendline = 3.5095 - 0.0009x Å, where x is %Mg.

16

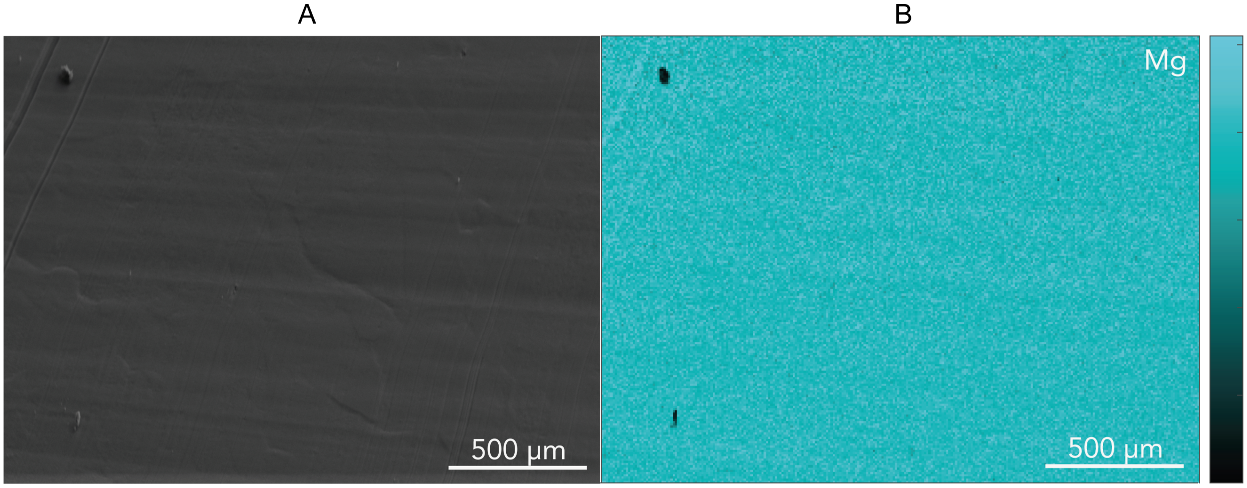

Supplementary Figure 2. Physiochemical characterization of lithium 5% Mg alloy. (a) SEM image of Li+5at%Mg sample surface. (b) Corresponding energy dispersive x-ray spectroscopy (EDX) map of Li+5at%Mg sample after casting. Showing homogeneous Mg distribution throughout the sample.

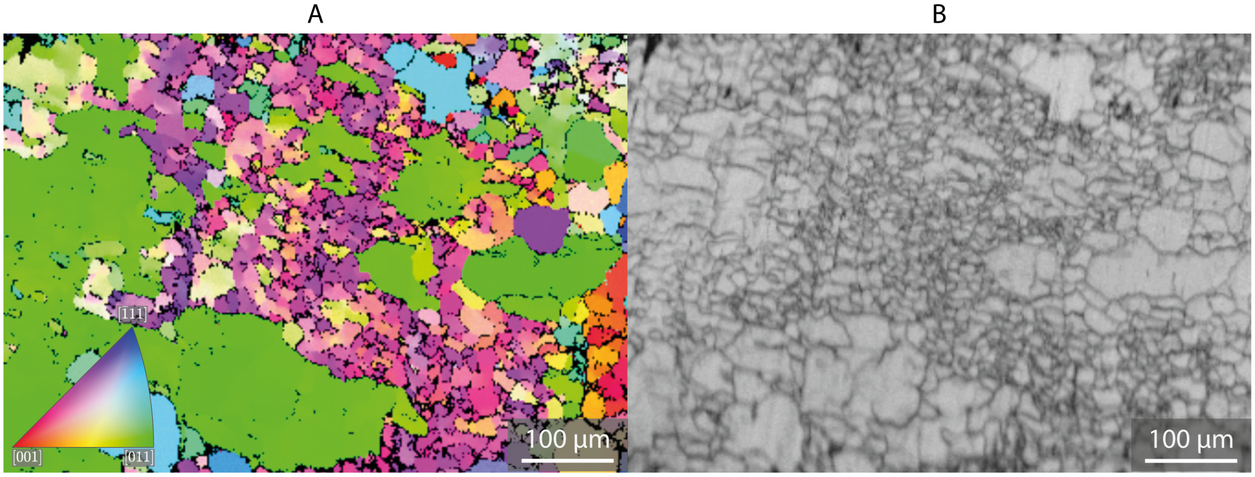

Supplementary Figure 3. EBSD map of dendritic Li+20at%Mg microstructure after casting. (a) EBSD map with inverse pole figure showing color-orientation relationship inset. Large regions with similar orientation are observed. (b) Band constrast of same region. Shows clear sub-grain boundaries within dendritic regions. Example of a dendritic solidification microstructure in Li+20at%Mg alloy, slow cooled overnight in a furnace from 450°C. Large regions can be seen in the orientation map, with grain boundaries visible in the band contrast map, and in unresolved orientation points. This microstructure is the result of dendritic solidification due to constitutional supercooling.

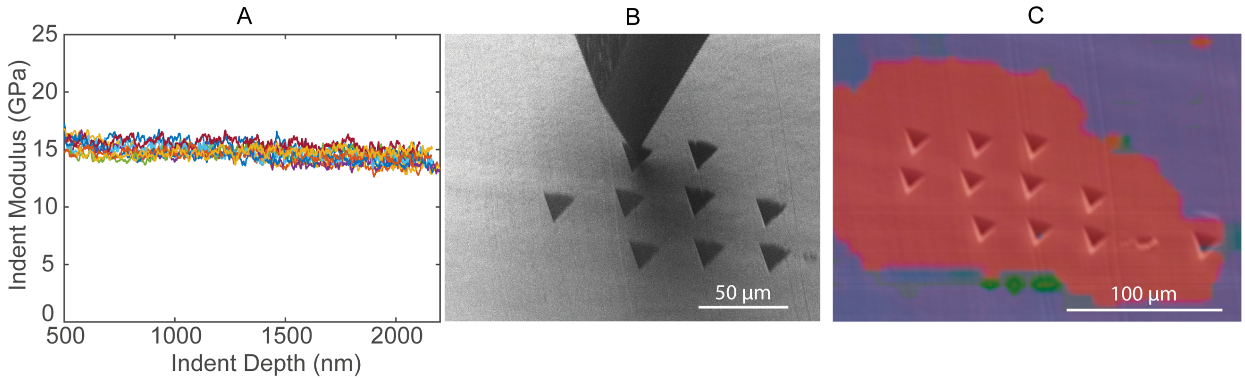

Supplementary Figure 4. Indentation of 10%Mg sample. (a) Indent modulus vs indent depth for indent array. (b) Secondary electron image during indentation, tilt corrected from 80 degrees. (c) EBSD IPF Z orientation map overlaid on secondary electron image of indent array, tilt corrected from 70 degrees. Grain boundary indent excluded from analysis.

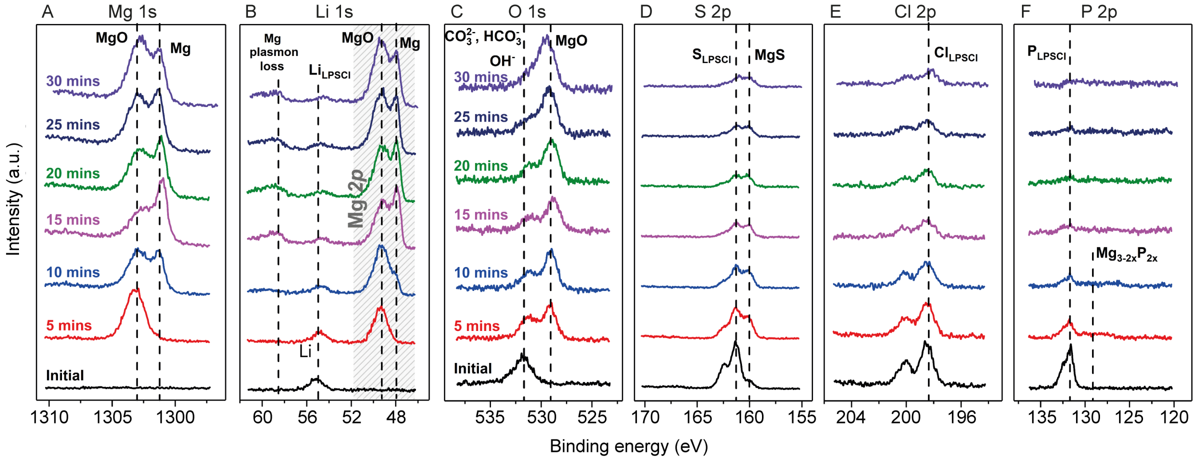

Supplementary Figure 5. XPS data of Mg sputtered in situ [1] onto Li_6PS_5Cl. Core-level XPS spectra collected at 5 min intervals during Mg sputtering conducted over a total period of 30 mins, for (a) Mg *1s*, (b) Li *1s*, (c) O *1s*, (d) S *2p*, (e) Cl *2p*, and (f) P *2p* transitions.

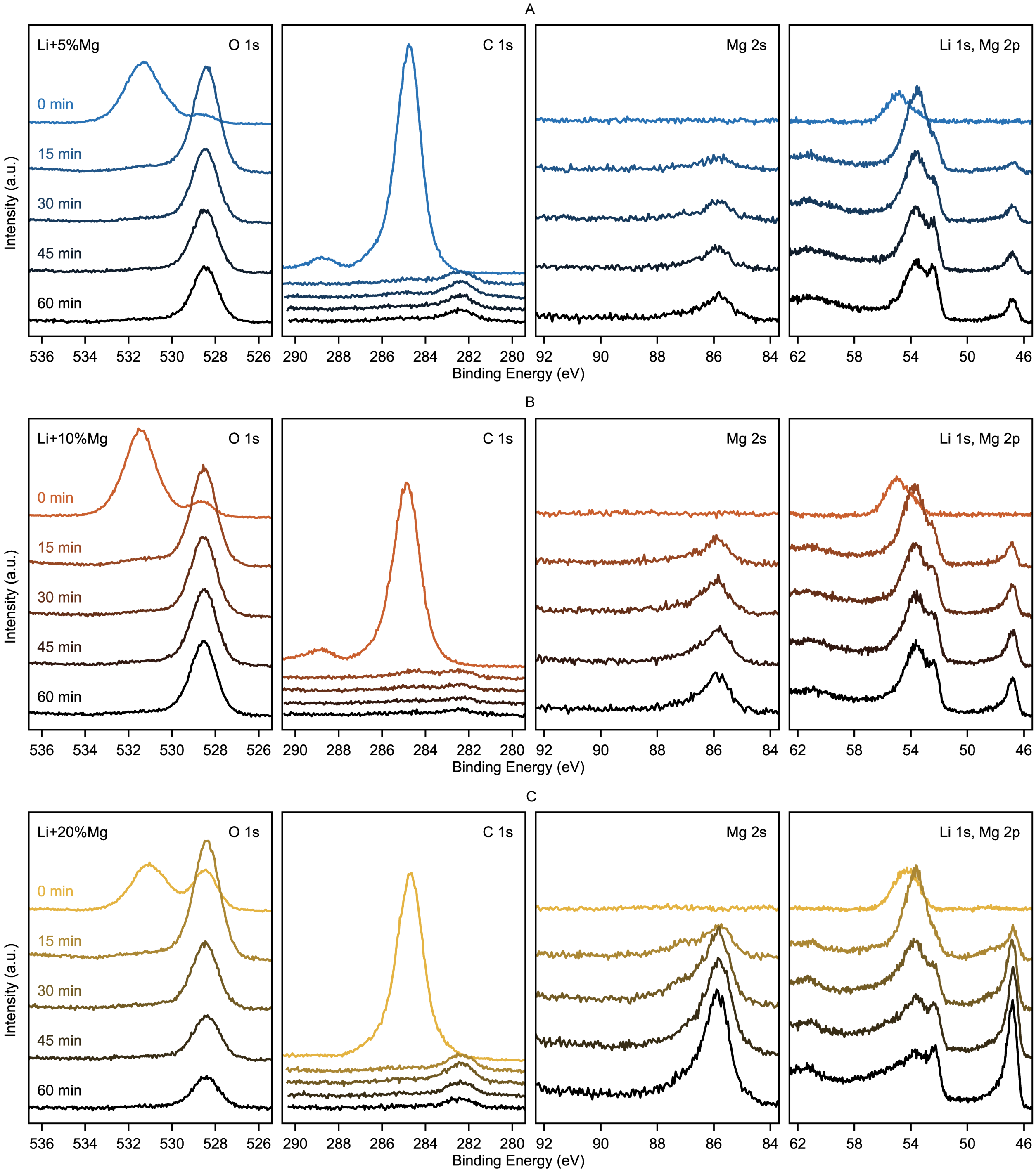

Supplementary Figure 6. XPS analysis to characterise native passivation layer on alloy surface, before cell assembly. Core-level XPS spectra for each alloy composition for O *1s*, C *1s*, Mg *2s*, Li *1s* and Mg *2p* conducted at 15-minute intervals during argon sputtering conducted over a total period of 60 mins. (a) Li+5%Mg, (b) Li+10%Mg, (c) Li+20%Mg.

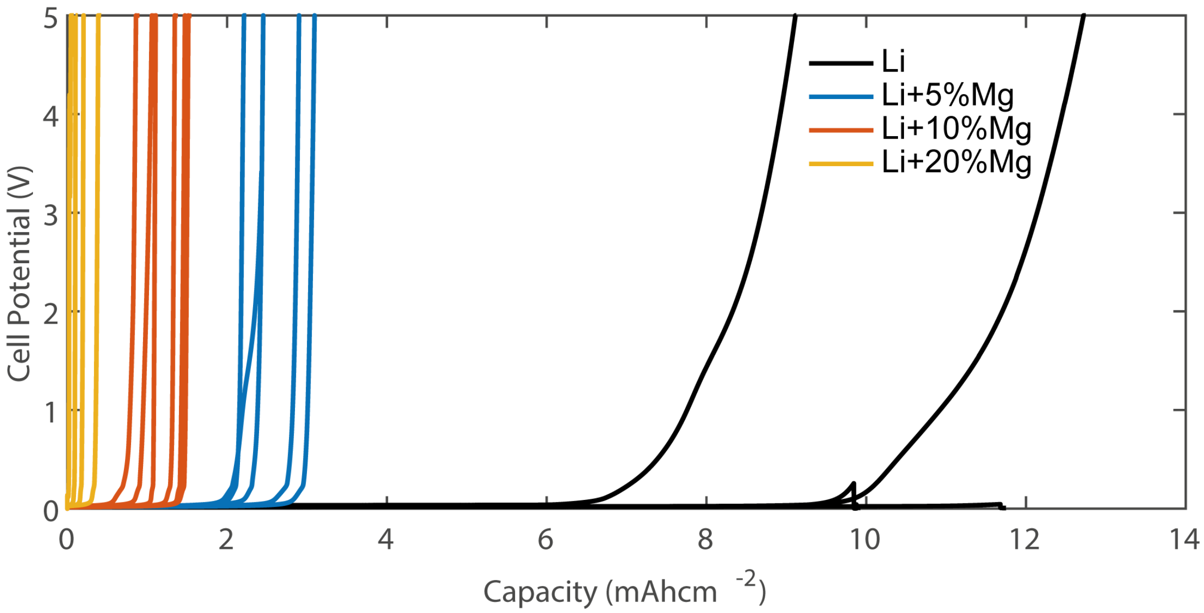

Supplementary Figure 7. Chronopotentiometric stripping of Li-Mg——Li-Mg symmetric cells showing cell potential as a function of areal capacity at 0.31 mAcm^{-2}, 30°C, 5 MPa. Selected representative data reproduced in main text.

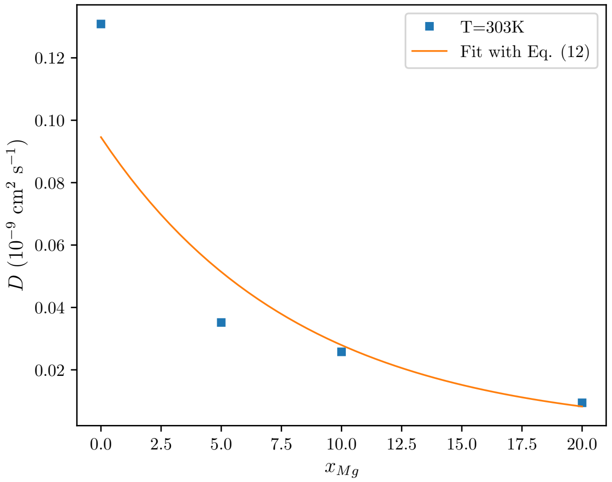

Supplementary Figure 8. Estimated values of the diffusion coefficient at T = 303 K as a function of the alloy composition. The symbols are values obtained by extrapolating Arrhenius relation and reported in Table 3. The line represents the fitting of these values using Eq. (12).

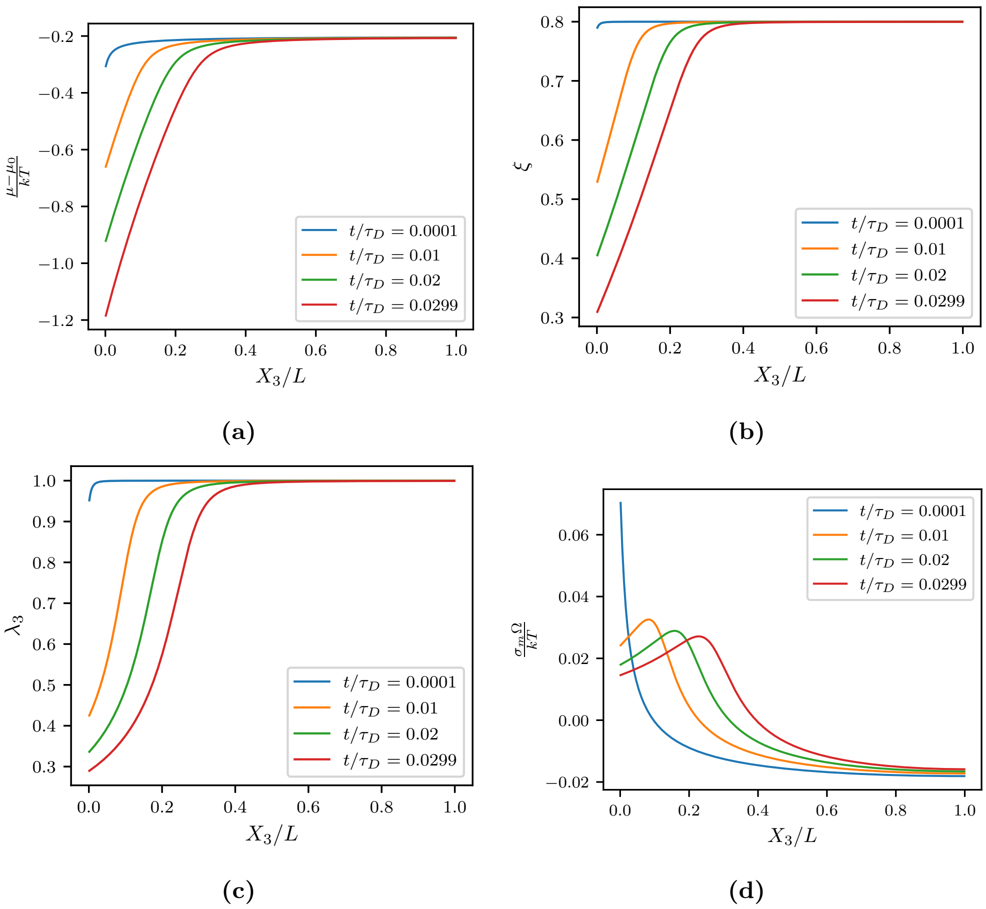

Supplementary Figure 9. Predicted profiles of (a) normalised chemical potential, (b) composition, (c) stretch and (d) normalised mean stress during stripping of a Li-Mg alloy with 80 at% Mg. In the simulations, L = 10 µm, D = 0.0082 × 10^{-9} cm^2 s^{-1} and P = 5 MPa.

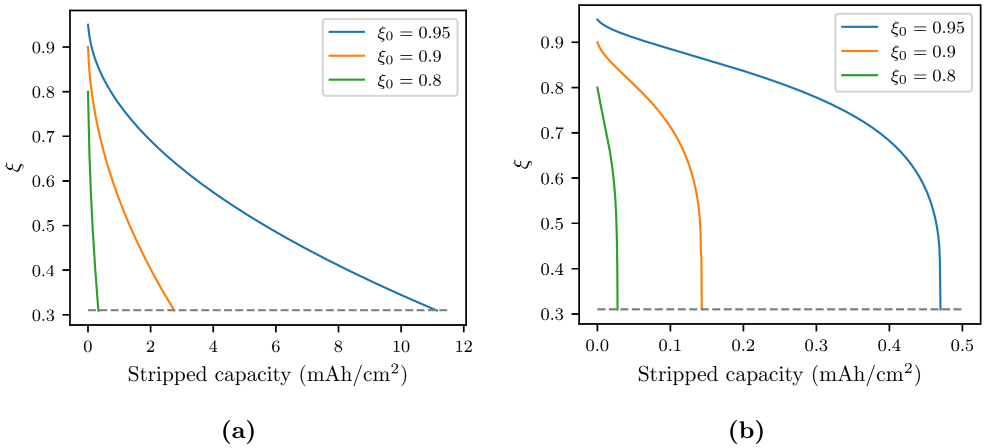

Supplementary Figure 10. Evolution of surface composition ξ(0, t) as a function of stripped capacity assuming (a) a constant diffusion coefficient calculated or (b) a variable diffusion coefficient, described by Eq. (12). Simulations are terminated when the composition reaches the critical value ξ = 0.31.

%Mg	n	stdev(n)	$\alpha^{1/n}$	stdev($\alpha^{1/n}$)	α
0	6.26	0.497	1.56×10^{-7}	2.83×10^{-8}	2.29×10^{-43}
5	7.78	0.746	5.98×10^{-8}	1.55×10^{-8}	6.59×10^{-57}
10	5.90	1.45	4.73×10^{-8}	2.05×10^{-8}	6.08×10^{-44}
20	4.24	0.335	8.96×10^{-9}	1.22×10^{-9}	7.48×10^{-35}

Supplementary Table 1. Fitted creep parameters with changing magnesium content.

	^{6}Li diffusion coefficient ($\times10^{-9}$ cm^{2}s^{-1})			
Temperature (°C)	Lithium	5% Mg	10% Mg	20% Mg
373.1	4.04(57)		1.26(14)	
383.1	5.75(24)	3.36(90)	2.18(19)	
393.1	8.83(37)	4.99(13)	3.00(10)	1.96(19)
403.1	11.9(11)	7.68(16)	4.60(16)	
413.1		11.65(22)		4.57(30)
423.1				6.41(36)
433.1				10.6(40)

Supplementary Table 2. Diffusion coefficients measured with 6Li PFG-NMR.

Alloy	$\alpha^{1/n}$ (Pa^{-1} s$^{-1/n}$)	n	D ($\times10^{-9}$ cm^2 s^{-1})
Li	1.56×10^{-7}	6.26	0.1309
5%	5.98×10^{-8}	7.78	0.0352
10%	4.73×10^{-8}	5.90	0.0258
20%	8.96×10^{-9}	4.24	0.0094

Supplementary Table 3. Creep and diffusion parameters of Li-Mg alloys used in the numerical simulations. Creep properties were directly measured by indentation. Diffusion coefficients at T = 303 K were calculated by fitting Arrhenius equation to experimental data reported in Table 2 for each alloy composition and extrapolating to room temperature. In simulations with variable diffusion coefficients, these values were further fitted using Eq. (12) to calculate the diffusion coefficient at any alloy composition.

Notes

Supplementary note 1 - XRD

XRD was performed on samples of the 5, 10 and 20% magnesium alloys with a Rigaku Miniflex. To create the samples, thin flakes were cut from the ingots using a microtome blade and mounted using petroleum jelly on a silicon single-crystal sample holder. The raw XRD data is shown in Supplementary Fig. 1A. There is a peak shift due to the change in lattice parameter with the magnesium content. Each peak was fitted with a pair of Gaussian-Lorentzian sum peaks in Matlab with a constant background, the {211} peak is plotted in Supplementary Fig. 1B as an example. The main source of errors in the measurement of peak position is the displacement of the sample surface from the diffractometer axis. To correct for this, the lattice parameters calculated from each fitted and indexed peak centre were plotted against $\cos^2\vartheta/\sin\vartheta$ and fit with a linear trend as shown in Supplementary Fig. 1C for Li+5%Mg. This trend extrapolated to zero (large theta), gives the corrected lattice parameter. These lattice parameters from the Kα1 and Kα2 peaks were averaged to give the fitted lattice parameters in Supplementary Fig. 1A, with the error bars showing the difference between the two values. The trend is linear, with a relationship of a = 3.5095 - 0.0009x Å, where x is %Mg.

Supplementary note 2 - XPS Mg in-situ sputtering

To investigate the possible reaction products formed from the presence of magnesium within the alloy electrodes, pure magnesium metal was sputtered in-situ [1] onto an argyrodite pellet using an Ar+ ion gun and the evolving interphase was characterised using x-ray photoemission spectroscopy (XPS). Supplementary Fig. 5A-B show core-level XPS spectra for the Mg *1s* and Li *1s* transitions from the surface of the pellet, at various intervals during etching of a magnesium foil. The initial oxygen *1s* spectra reveals the presence of surface contamination comprising typically of carbonates, bicarbonates and hydroxides, whose intensity reduces with increased Mg sputtering. After just five minutes of sputtering (at an Ar^+ ion accelerating voltage of 4 kV) a peak characteristic of magnesium oxide appears at a binding energy (B.E.) of 1303 eV in the Mg *1s* spectra, with an equivalent peak appearing in the oxygen *1s* spectra at a B.E. of 529 eV [2]. Despite the very low oxygen content of the XPS chamber, the metallic magnesium layer deposited can easily react to form MgO [3, 4]. Further magnesium sputtering results in the appearance of another Mg *1s* peak at a lower B.E. ($\sim$1301 eV) characteristic of metallic magnesium, that increases in intensity as sputtering proceeds for longer periods. The electrochemical potential of pure magnesium is well below the stability window of Li_6PS_5Cl, so it will be reduced with magnesium deposition. Li_6PS_5Cl reacts with lithium to form Li_2S, suggesting that the S^{2-} is weakly bound within the ${PS_4}^{3-}$ tetrahedron. [5]. The growth of a peak at the lower B.E. shoulder ($\sim$160 eV) in the S *2p* spectra (Fig. 5D) could be indicative of the formation of MgS [6] but is indistinguishable from an Li_2S peak. The same is true in the Mg *1s* spectra, where any MgS peak would be at a B.E. similar to that of MgO [7, 8], but the growth of this peak after 15 minutes perhaps suggests the formation of MgS. No significant changes in the Cl *2p* spectra were observed (Fig. 5E), likely due to the proximity in B.E. values for the Cl in argyrodite and that in $MgCl_2$, as has been noted previously for LiCl [5]. Lastly, in the P *2p* spectra (Fig. 5F) there is a broad feature between 126-130 eV, ascribed to the formation of sub-phosphides which has equivalently been observed in the case of lithium sputtering [9], is present in very low intensities between five and ten minutes of sputtering.

Supplementary note 3 - Coupled creep-diffusion model

Theory

We formulate a continuum model for coupled creep and diffusion in a layer of Li-Mg alloy of thickness L, building a recently-developed continuum framework for inelastic deformations coupled to diffusion in mixtures [10]. The in-plane dimensions of the layer are much larger than the out-of-plane dimension, so that diffusion and deformation only take place in the out-of-plane, X_3-direction. We only consider the diffusion of Li atoms, and use the Mg atoms as markers to track the alloy deformation. The principal stretches are

given by:

$$\lambda_3(X_3, t) = \frac{\partial x_3(X_3, t)}{\partial X_3}, \quad \lambda_1 = \lambda_2 = 1 \tag{1}$$

where x_3 is the current coordinate of a marker with coordinate X_3 in the reference configuration. The swelling ratio is given by $J = \lambda_1\lambda_2\lambda_3 = \lambda_3$. We also introduce the true principal strains $\varepsilon_i = \log(\lambda_i)$, and the principal deviatoric strains: $e_i = \varepsilon_i - \mathrm{tr}(\boldsymbol{\varepsilon})/3$. The layer is subjected to a constant stack pressure P in the X_3 direction. Mechanical equilibrium then requires that $\sigma_3(X_3, t) = -P$, where σ_3 is the normal Cauchy stress in direction X_3. Since deformations are prevented in the in-plane directions, the layer is in a state of equibiaxial stress: $\sigma_1(X_3, t) = \sigma_2(X_3, t)$.

Let $C(X_3, t)$ be the nominal concentration of Li (number of Li atoms per unit volume in the reference configuration). Alternatively, the local composition can be described by the Li atomic fraction $\xi(X_3, t)$ (number of Li atoms per total number of atoms):

$$\xi = \frac{\Omega C}{J} \tag{2}$$

where Ω is the volume per lithium atom. Eq. (2) holds under the condition that Mg and Li have similar atomic volumes. In the initial state, $J(X_3, 0) = 1$ and $\xi(X_3, 0) = \Omega C(X_3, 0) \equiv \Omega C_0 \equiv \xi_0$, where C_0 and ξ_0 are the initial nominal concentration and composition, assumed uniform through the thickness. Alloys with 5%, 10% and 20% Mg respectively correspond to $\xi_0 = 0.95$, 0.9 and 0.8. The evolution of composition in the alloy over time is dictated by the equation of conservation of lithium:

$$\frac{\partial C}{\partial t} = -\frac{\partial J_3}{\partial X_3} \tag{3}$$

where $J_3(X_3, t)$ is the nominal diffusion flux in the X_3-direction (number of Li atoms per unit area in the reference configuration per time). Boundary conditions are set as follows. The stripping flux $\bar{I}$ is prescribed at the electrode-electrolyte interface: $J_3(0, t) = \bar{I}$, and the diffusion flux vanishes at the electrode-current collector interface: $J_3(L, t) = 0$.

We make the assumption of molecular incompressibility of the solution, that is, the volume of the solution is independent of stress. The swelling ratio of a material element associated with a Mg marker is then set by the composition of the material element:

$$J = 1 + \Omega(C - C_0) \tag{4}$$

Eq. (4) states that a material element associated with a marker shrinks as Li atoms are being stripped. The kinematic constraint (1) on the lateral deformation couples the deviatoric and volumetric strain rates. By definition of e_1, we have:

$$\dot{e}_1 = -\frac{1}{3}\frac{\dot{\lambda}_3}{\lambda_3} = -\frac{1}{3}\frac{\Omega\dot{C}}{\lambda_3} \tag{5}$$

where we have also used the incompressibility condition (4). This relation expresses that the shrinking due to the stripping of lithium ($\dot{C} < 0$) must be accommodated by a deviatoric strain rate ($\dot{e}_1 > 0$), so that $\dot{\varepsilon}_1 = 0$.

We neglect elastic deformations, so that the deviatoric strain rates are only due to creep. Introduce the principal deviatoric stresses $s_i = \sigma_i - \mathrm{tr}(\boldsymbol{\sigma})/3$. Deviatoric stresses and strain rates are related by the associative creep flow rule:

$$\dot{e}_i = \frac{3}{2}\dot{p}\frac{s_i}{\sigma_{eq}}, \quad i = 1, 2, 3 \tag{6}$$

where $\dot{p} = ((2/3)\dot{e}_i\dot{e}_i)^{1/2}$ is the equivalent (accumulated) plastic strain rate, and $\sigma_{eq} = ((3/2)s_is_i)^{1/2}$ is the von Mises stress (summation over repeated indices is used). For equibiaxial loading, one easily verifies that $\dot{p} = 2|\dot{e}_1|$ and $\sigma_{eq} = 3|s_1|$. The flow rule (6) thus simply means that $\mathrm{sign}(\dot{e}_1) = \mathrm{sign}(s_1)$. The accumulated plastic strain is related to the von Mises stress by the power-law:

$$\dot{p} = \alpha\sigma_{eq}^n \tag{7}$$

where α and n are the creep pre-factor and exponent, respectively. Combining Eqs (5) and (7) gives:

$$\sigma_{eq} = \left(\frac{\dot{p}}{\alpha}\right)^{1/n} = \left(\frac{2}{3\alpha}\frac{\Omega|\dot{C}|}{\lambda_3}\right)^{1/n} \tag{8}$$

This equation sets the equivalent stress required to drive creep and accommodate the volume change due to stripping lithium.

The diffusion of lithium in the alloy is assumed to be driven by the gradient in chemical potential. Let j_3 be the true diffusion flux (number of Li atoms per unit area in the current configuration per time), we adopt a diffusion model of the form:

$$j_3 = -\frac{cD}{kT}\frac{\partial\mu}{\partial x_3} \tag{9}$$

where $c = C/J$ is the true Li concentration (number of Li atoms per unit volume in the current configuration), μ is the chemical potential of Li in the alloy, D is the diffusion coefficient, k is Boltzmann's constant, and T is the absolute temperature. Rewriting Eq. (9) in terms of nominal quantities gives:

$$J_3 = -\frac{CD}{kT}\frac{1}{\lambda_3^2}\frac{\partial\mu}{\partial X_3} \tag{10}$$

The chemical potential is taken of the following form:

$$\mu = \mu_0 + kT\log(\xi) - \Omega\sigma_m \tag{11}$$

where μ_0 is the chemical potential of pure Li in the absence of stress and $\sigma_m = \mathrm{tr}(\boldsymbol{\sigma})/3$ is the mean stress. The stress contribution to chemical potential arises from the incompressibility constraint (4) which couples the Li change in concentration to the volume change. For a derivation of expression (11) from thermodynamics principles, see Ref. [10]. For equibiaxial loading, the mean stress is related to the deviatoric stress and stack pressure by $\sigma_m = 2s_1 - P$. Recalling that $\sigma_{eq} = 3|s_1|$, the mean stress can thus be calculated as a function of the concentration and concentration rates via Eq. (8).

Numerical solution

We numerically solved the 1D diffusion equation (3) for the unknown field $\mu(X_3, t)$ using the Finite Element Method (FEM) with linear shape functions for the spatial discretisation, combined with a fully-implicit time-integration scheme. Details on the FE formulation and implementation can be found in Ref. [10]. At each integration point, the current concentration is calculated from the chemical potential using the state law (11), where the Li composition ξ and the mean stress σ_m are expressed as functions of the concentration and concentration rates. In practice, Eq. (8) is solved numerically using Brent's method as implemented in the scipy.optimize package of Python. The system of nonlinear equations arising from the FE discretisation is solved iteratively using an in-house implementation of the Newton-Raphson method. The numerical domain was discretised using 400 elements, and the physical length of the domain was adjusted to capture local phenomena near the interface, with a maximum length equal to 100 µm.

Material parameters used in the simulations were the following. The volume per Li atom was determined from the lattice parameter for pure Li (3.51 Å), giving $\Omega = 2.16 \times 10^{-29}$ m^3 per Li atom. Creep parameters α and n for a given initial alloy composition were set to their value identified from indentation creep tests and are reported in Table 3. Creep parameters were kept constant during stripping for simplicity, since we found that creep parameters do not impact the stripping performance in the simulations. The diffusion coefficient of Li at 303K was determined from Arrhenius relation with activation energy and pre-factor fitted from experimental data reported in Table 2. Extrapolated diffusion coefficient values at 303K are reported in Table 3. These values were further fitted using the empirical relation:

$$D(x_{Mg}) = a\exp(-bx_{Mg}) \tag{12}$$

where $x_{Mg} = 100(1-\xi)$ is the Mg atomic fraction in % and $a = 9.4584 \times 10^{-15}$ m^2/s and $b = 0.122$. The fitting relation is compared to the extrapolated values based on Arrhenius relation in Supplementary Fig. 8. Two scenarios were considered in the numerical simulations. In the first scenario, the diffusion coefficient of Li in the alloy was kept constant to its initial value. In the second scenario, the diffusion coefficient was assumed to depend on the current alloy composition according to Eq. (12). These two scenarios are referred to as "constant diffusivity" and "variable diffusivity" in the main text.

Prescribed current boundary conditions were specified on either side of the domain. At the interface with the electrolyte ($X_3 = 0$), the stripping Li flux $\bar{I}$ (number of Li atoms per area and per time) was calculated based on the the experimental stripping current of 0.31 mA cm^{-2} as: $\bar{I} = 3.1/e$ m^{-2} s^{-1}, where $e = 1.602 \times 10^{-19}$ C is the electron charge. Zero-current conditions were imposed at the other end of the simulation domain ($X_3 = L$). Uniform compositions of either $\xi_0 = 0.99$, 0.95, 0.9 and 0.8 were used as initial conditions. Simulations were interrupted when the alloy composition reached the critical composition $\xi_c = 0.31$ of Li at the electrolyte-electrode interface, corresponding to the saturation limit (69 at% Mg) of the BCC alloy phase.

Simulation results

Representative simulation results for a Li-Mg alloy with 20 at% Mg are shown in Supplementary Fig. 9. Predicted profiles of (a) normalised chemical potential $\frac{\mu-\mu_0}{kT}$, (b) composition ξ, (c) stretch λ_3 and (d) normalised mean stress $\frac{\sigma_m\Omega}{kT}$ are shown at different simulation times normalised by the characteristic diffusion time $\tau_D = L^2/D$, with $L = 10$ µm and $D = 0.0082 \times 10^{-9}$ cm^2 s^{-1} (predicted diffusion coefficient using Eq. (12) for $x_{Mg} = 20\%$). The profile at $t/\tau_D = 0.0299$ corresponds to the time when the critical composition $\xi = 0.31$ is reached on the interface $X_3 = 0$. Simulations are run assuming that the diffusion coefficient remains constant during stripping, and the stack pressure is 5 MPa ($\frac{P\Omega}{kT} = 0.026$). The removal of lithium is accompanied by significant stretch $J = \lambda_3 < 1$ near the interface due to the molecular incompressibility assumption (4). Creep flow develops to preserve zero-deformation in the lateral direction, requiring the development of a transient heterogeneous stress field. While the mean stress far from the interface is compressive and equal to the stack pressure, a tensile mean stress develops near the interface.

The maximum stripped capacity is reached when the surface composition reaches the critical value $\xi = 0.31$. The evolution of the surface composition as a function of the stripped capacity is shown in Supplementary Fig. 10 for three alloy compositions, assuming (a) constant diffusion coefficient or (b) variable diffusion coefficient, both described by Eq. (12). In the first scenario, the predicted capacity overestimates the experimental capacity, while in the second scenario it underestimates the experimental capacity (see Fig. 5C in the main text). Simulations results were obtained under a stack pressure $P = 5$ MPa. However, the same capacity values were found for stack pressure values in the range 0-10 MPa. We have also verified that the stripped capacity is largely independent of the creep parameters used. Overall, our numerical results suggest that the maximum capacity is governed by the diffusion coefficient of lithium in the alloy, rather than by the effect of stress on diffusion.

Supplementary Note 4 - Elastic anisotropy indentation measurement.

The sample surface normal to an elastic half space ($\hat{\mathbf{u}}$) in the Cartesian system with its three axes along the principle <100> axes of the crystal, is calculated from the Euler angles from EBSD as in Equation (13). Next, the integral in Equation (14) is computed where gamma is the angle between a vector $\mathbf{t}$, which lies in the half-space boundary and starts at the origin, and some fixed datum in the half-space boundary. The matrix β is itself defined by Equation (15), with $\mathbf{m}$ and $\mathbf{n}$ being two perpendicular vectors to $\mathbf{t}$, such that they form a right-handed cartesian set, with the integral over phi summing the contributions for all orientations of $\mathbf{m}$ and $\mathbf{n}$ in the plane. The matrices of type ($\mathbf{ab}$) are defined by the final Equation (16), with C being the elastic stiffness tensor. The indices are converted by the convention $11 \rightarrow 1$, $22 \rightarrow 2$, $33 \rightarrow 3$, $12 \rightarrow 4$, $23 \rightarrow 5$, $13 \rightarrow 6$. α is the cosine of the direct angle between the surface normal and the axis of the subscript. Einstein notation is used throughout. A corrective factor of 1.06 is applied to account for the triangular shape of the Berkovich tip [11]. The calculated indentation modulus solely depends on the orientation of the

sample surface normal relative to the crystal lattice, and the stiffness matrix of the material. This numerical calculation was implemented in a Matlab script, allowing the generation of a predicted measured modulus surface in orientation surface from given stiffness coefficient values. The set of values for C_{11}, C_{12} and C_{44} that gave the least squares fit to the orientation-modulus data were found using a Nelder-Mead algorithm, using a tetragonal simplex, implemented in the fminsearch function in MATLAB with a threshold value for all three of the stiffness components of 0.1.

$$\hat{\mathbf{u}} = \begin{bmatrix} \sin(\psi)\sin(\Phi) \\ -\cos(\psi)\sin(\Phi) \\ \cos(\Phi) \end{bmatrix} \tag{13}$$

$$M = 1.06 \times 16\pi^2 \left(\int_0^{2\pi} \alpha_m \beta_{km}^{-1}(\gamma) \alpha_k d\gamma \right)^{-1} \tag{14}$$

$$\beta_{km}(\mathbf{t}) = \frac{1}{8\pi^2} \int_0^{2\pi} \{(\mathbf{mm})_{js} - (\mathbf{mn})_{jk} (\mathbf{nn})_{kr}^{-1} (\mathbf{nm})_{rs}\} d\psi \tag{15}$$

$$(\mathbf{ab})_{jk} = a_i C_{ijkm} b_m \tag{16}$$

References

1. Narayanan, S. *et al.* Effect of current density on the solid electrolyte interphase formation at the lithiumLi6PS5Cl interface. *Nature Communications* **13,** 7237 (2022).
2. Otto, S. K. *et al.* In-Depth Characterization of Lithium-Metal Surfaces with XPS and ToF-SIMS: Toward Better Understanding of the Passivation Layer. *Chemistry of Materials* **33,** 859–867 (2021).
3. Wood, K. N. *et al.* Operando X-ray photoelectron spectroscopy of solid electrolyte interphase formation and evolution in Li2S-P2S5 solid-state electrolytes. *Nature Communications* **9,** 2490 (2018).
4. Gibson, J. S. *et al.* Gently does it!: in situ preparation of alkali metal-solid electrolyte interfaces for photoelectron spectroscopy. *Faraday Discussions* **236,** 267–287 (2022).
5. Wenzel, S., Sedlmaier, S. J., Dietrich, C., Zeier, W. G. & Janek, J. Interfacial reactivity and interphase growth of argyrodite solid electrolytes at lithium metal electrodes. *Solid State Ionics* **318,** 102–112 (2018).
6. Salama, M. *et al.* Metal-Sulfur Batteries: Overview and Research Methods. *ACS Energy Letters* **4,** 436–446 (2019).
7. Lahtinen, J., Vaari, J., Talo, A., Vehanen, A. & Hautojärvi, P. Studies of Mg-O overlayers on Co(0001): growth mode and CO chemisorption properties. *Surface Science* **245,** 244–254 (1991).
8. Vinayan, B. P. *et al.* Performance study of magnesium-sulfur battery using a graphene based sulfur composite cathode electrode and a non-nucleophilic Mg electrolyte. *Nanoscale* **8,** 3296–3306 (2016).
9. Schwietert, T. K. *et al.* Clarifying the relationship between redox activity and electrochemical stability in solid electrolytes. *Nature Materials* **19,** 428–435 (2020).
10. Brassart, L., Liu, Q. & Suo, Z. Mixing by shear, dilation, swap, and diffusion. *Journal of the Mechanics and Physics of Solids* **112,** 253–272 (2018).
11. Vlassak, J. J. & Nix, W. Measuring the Elastic Properties of Materials By Means of Indentation. *J. Mech. Phys. Solids* **42,** 1223–1245 (1994).
